# Is the CALLY Index the Best Tool for Predicting Survival in Patients with Primary Soft Tissue Sarcomas?

**DOI:** 10.3390/diagnostics16162559

**Published:** 2026-08-14

**Authors:** Tomoki Nakamura, Tomohito Hagi, Yumi Matsuyama, Hironori Date, Masahiro Hasegawa

**Affiliations:** Department of Orthopaedic Surgery, Graduate School of Medicine, Mie University, Tsu 514-8507, Japan

**Keywords:** CALLY index, modified Glasgow prognostic score, prognostic nutrition index, nutrition, inflammation, soft tissue sarcoma

## Abstract

**Background/Objectives**: To elucidate the role of the CALLY index in 172 adult patients with primary soft tissue sarcoma (STS) and to evaluate whether the CALLY index is superior to the modified Glasgow prognostic score (mGPS) and prognostic nutrition index (PNI). **Methods**: The study cohort included 100 men and 72 women with a mean age of 63 years. Receiver operating characteristic (ROC) curve analysis was performed to determine the threshold value of the CALLY index for identifying the risk of identifying oncological events at 2 years. **Results**: ROC analysis identified 2.07 as the threshold of the CALLY index for predicting the risk of identifying oncological events at 2 years. The 5-year disease-specific survival (DSS) rate was 50.9% in patients with a lower CALLY index compared to 84% in those with a higher CALLY index. All patients with mGPS scores of 1 or 2 had lower CALLY indices, whereas all patients with a higher CALLY index had an mGPS score of 0. Patients with a lower CALLY index and mGPS of 0 had better DSS than those with a lower CALLY index and mGPS of 1 or 2. No substantial differences in DSS and disease-free survival (DFS) were observed between patients with a higher CALLY index and those with a lower CALLY index and an mGPS of 0. **Conclusions:** The CALLY index is a useful marker for predicting oncological outcomes in patients with primary STS. In particular, the CALLY index was a statistically significant prognostic variable for survival in patients with UPS and DDLPS individually. However, we suggest that the mGPS remains a reliable system for identifying patients with primary STSs who are at a high risk of death and relapse because we showed the significant difference for survival and event-free survival between the patients with mGPS of 1 and 2.

## 1. Introduction

Soft tissue sarcoma (STS) is a rare heterogeneous tumor. The incidence of STS is less than 6 per 100,000 cancer cases, accounting for 1–2% of all cancer cases in adults [1]. Although there are more than 50 histologic subtypes, the standard treatment in patients with primary non-metastatic STS remains wide local resection, except for round-cell sarcoma such as extra-skeletal Ewing sarcoma, and rhabdomyosarcoma. It must be carried out by a surgeon specifically trained in the treatment of this disease within a sarcoma-specific center. The standard surgical treatment is a complete tumor resection with R0 margin [2]. This implies removing the tumor in a single specimen with a rim of normal tissue around it. Neoadjuvant or adjuvant radiotherapy is typically added to surgery as part of the standard treatment of high-grade STS [2]. Radiotherapy is not commonly indicated in the case of a truly compartmental resection of a tumor entirely contained within the compartment. Also, radiotherapy may be omitted after multidisciplinary discussion, considering risk factors for local recurrence, including surgical margins, tumor size, depth, and histological type. Perioperative chemotherapy can be proposed for fit patients affected by disease at high risk of death [2]. Although complete local treatment is performed, lung metastasis develops in 20–50% of STS patients, and the subsequent prognosis is poor. Conventional prognostic factors such as age at diagnosis, tumor size, histological grade, and tumor depth are useful general risk assessment tools for determining the prognosis, surgical strategy, and adjuvant management of patients with STS [3,4]. In addition to conventional prognostic factors, non-invasive prognostic biomarkers identified using a range of standard test results have attracted attention because they are cost-effective, easy to measure, and familiar to physicians [5,6,7]. Immune system, nutritional condition, and inflammation should be deeply associated with cancer progression. Therefore, we noted the prognostic tools which reflect the nutritional status, inflammatory status, and immune-system status in patients with STS. The prognostic nutritional index (PNI), calculated based on a combination of serum albumin and absolute lymphocyte count, has been used as a prognostic tool for survival in cancer patients [8]. Inadequate nutritional conditions and immunosuppression, which are commonly found in patients with cancers, also adversely affect clinical outcome and are reflected in biochemical indicators of serum albumin and lymphocytes. Reduced levels of these indicators not only indicate malnutrition and reduced immunocompetence [8]. Recently, the relationship between the survival and PNI was reported in the patients with STS, especially for retroperitoneal STS [9,10,11]. The modified Glasgow Prognostic Score (mGPS) underscores the value of C-reactive protein (CRP) as a standalone prognostic factor and recognizes hypoalbuminaemia’s negative impact only when CRP is elevated. mGPS is now widely applied for the prognostic tool for the patients with many types of cancer including STS and is backed by substantial evidence of its prognostic value. Recently, a large international multicenter study validated mGPS as an independent prognostic indicator for disease-free survival (DFS) and overall survival (OS) in patients with a localized resectable STS [12,13,14,15]. Thus, the PNI is considered to reflect the immune, nutritional status of patients with cancer, and mGPS is considered to reflect the nutritional, and inflammatory status of patients with cancer. Recently, the CALLY index has been shown to have prognostic potential in oncology, which reflects the immune, nutritional, and inflammatory status of patients with cancer [16,17,18]. The CALLY index, based on CRP and albumin levels and absolute lymphocyte counts, has shown potential for predicting cancer prognosis and guiding treatment [16,17,18]. However, there are no reports on the relationship between the CALLY index and STS. Therefore, we investigated the role of the CALLY index in 172 adult patients with primary STS. Furthermore, we evaluated the superiority of the CALLY index over the modified Glasgow prognostic score (mGPS) and prognostic nutrition index (PNI), as these indicators include CRP, albumin, and lymphocyte levels. Multivariate analysis showed that the CALLY index remained a significant predictor of DSS.

## 2. Materials and Methods

This study was conducted in accordance with the Strengthening the Reporting of Observational Studies in Epidemiology (STROBE) guidelines. Patients who underwent surgical resection for primary STS between January 2004 and December 2023 were included in this study. We excluded patients with metastasis at the initial presentation and those who underwent unplanned excision. Patients with well-differentiated liposarcoma (LPS) and dermatofibrosarcoma protuberances were also excluded. This study was approved by the Clinical Research Ethics Review Committee of Mie University Hospital, Japan (H2020-224) on 18 November 2020, and conducted in accordance with the Declaration of Helsinki. The need for informed consent was waived owing to the retrospective study design. Instead, the patients were allowed to opt out. The study cohort included 100 men and 72 women with a mean age of 63 years (range, 21–89 years) at the time of primary STS surgery. The mean follow-up period was 78 months. The minimum follow-up duration was 2 years, except for patients who experienced oncological events within 2 years. Blood samples were collected before treatment. The CALLY index was calculated using the formula: (serum albumin level (g/dL) × absolute lymphocyte count (n/μL))/C-reactive level (mg/dL) × 10,000. The modified Glasgow prognostic score (mGPS) was calculated as follows: patients with both hypoalbuminemia (<3.5 g/dL) and an elevated CRP level (>1.0 mg/dL) were allocated a score of 2. Those with elevated CRP levels were assigned a score of 1. The remaining patients were assigned a score of 0. The prognostic nutritional index (PNI) was calculated from preoperative laboratory parameters using the formula: 10× serum albumin level (g/dL) + 0.005× lymphocyte count (n/μL) in the peripheral blood. Blood samples were obtained from all patients before treatment. The primary purpose of this study was to elucidate the relationship between the CALLY index and DSS in 172 patients with primary STS who underwent surgical resection. The secondary purpose of this study was to elucidate whether the CALLY index is the best tool for predicting survival compared to the mGPS and PNI.

### Statistical Analysis

The clinicopathological factors were evaluated using the Mann–Whitney *U*-test and Kruskal–Wallis rank sum test (quantitative data), and chi square test (qualitative data). The correlations between quantitative data were tested using Spearman’s rank correlation analysis. DSS was defined as the time from the date of surgical resection for primary STS to the date of death or the last follow-up examination. Disease-free survival (DFS) was defined as the time from the date of surgical resection for primary STS to the date of local recurrence, distant metastasis, or last follow-up examination. Survival curves were constructed using the Kaplan–Meier method. Univariate analyses were conducted using the log-rank test for qualitative data or the Cox proportional hazards model (quantitative data) to compare survival rates. Multivariate analyses were performed using the Cox proportional hazards model. Values of *p* < 0.05 were included as variables in the multivariate analysis. Receiver operating characteristic (ROC) curve analysis was performed to determine the thresholds of the CALLY index and PNI for the risk of death at 2 years. All statistical analyses were performed using the EZR (version 1.70) graphical user interface (Saitama Medical Center, Jichi Medical University, Saitama, Japan) for R (version 4.20, R Foundation for Statistical Computing, Vienna, Austria), a modified version of R Commander designed to add statistical functions that are frequently used in biostatistics. *p* values < 0.05 were considered significant in all statistical analyses.

## 3. Results

### 3.1. Patient Characteristics

A total of 172 patients were included in this study (Table 1). The average tumor size was 8.5 cm (range, 2–30 cm). There were 39 and 133 patients of superficial and deep STS, respectively. The primary tumor sites were the thigh (*n* = 66), leg (*n* = 23), buttock (*n* = 15), chest wall (*n* = 15), upper arm (*n* = 12), back (*n* = 11), knee (*n* = 6), and others (*n* = 11). Three cases of retroperitoneal STS were included. Primary STS was histologically classified as follows: 39 undifferentiated pleomorphic sarcomas (UPS), 36 myxofibrosarcomas (MFS), 26 leiomyosarcomas (LMS), 17 myxoid liposarcomas (MLPS), 15 dedifferentiated liposarcomas (DDLPS), 9 malignant peripheral nerve sheath tumors (MPNST), 8 synovial sarcomas, and 22 tumors of other histological types. There were 23 and 149 patients of histologically low-grade and high-grade STS, respectively. All patients underwent surgical tumor resection for primary STS. Perioperative chemotherapy and radiotherapy were performed in 38 patients and 18 patients, respectively. Perioperative chemotherapy was given to those patients under 70 years with high-grade, deep and large (>5 cm) tumors. Radiotherapy was given to those patients with tumors in which an adequate margin could not be achieved and those in whom the margins contained tumor.

#### 3.1.1. Clinical Outcomes

**Figure 1:**
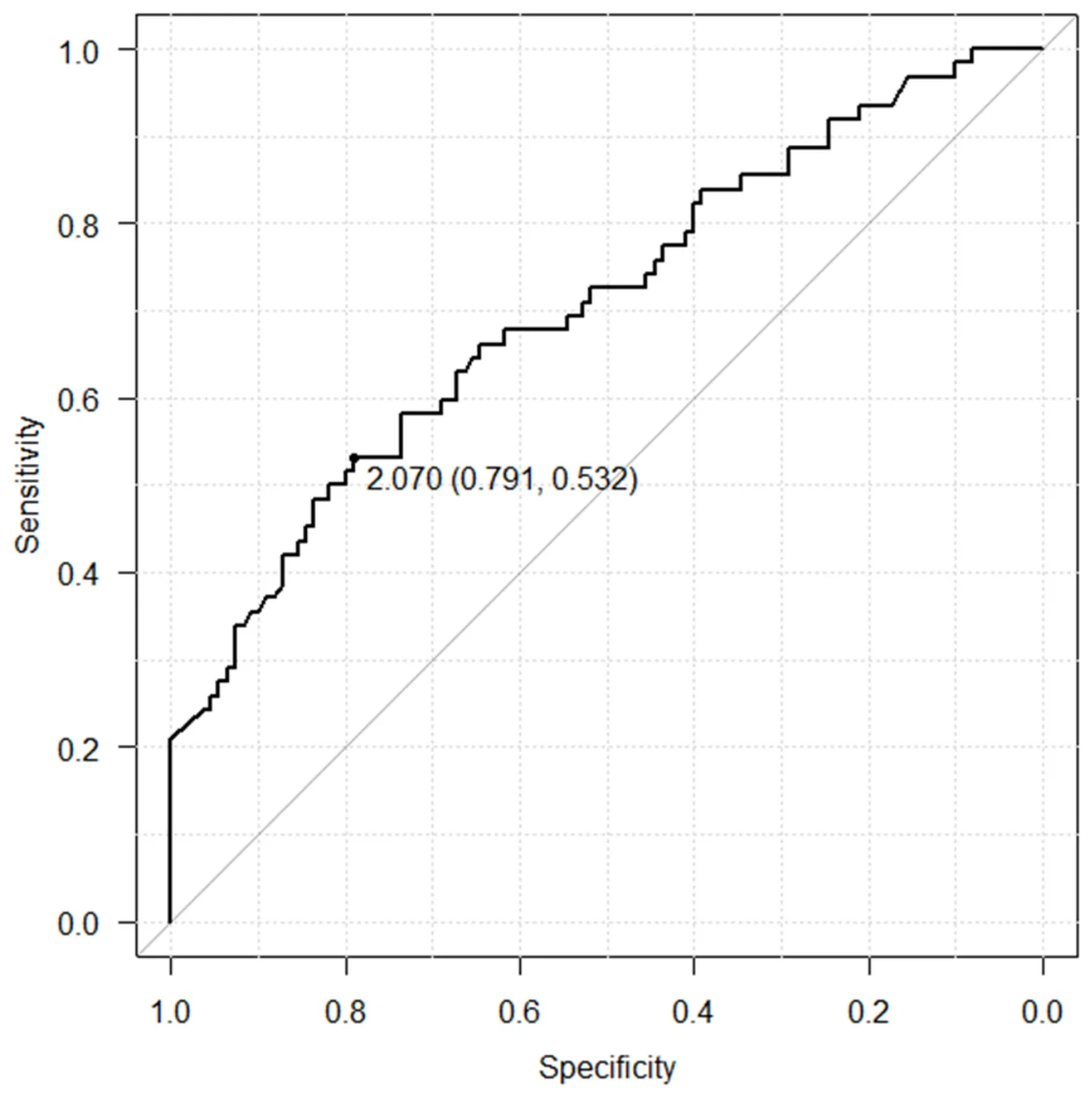
Receiver operative characteristic (ROC) analysis shows the threshold (2.07) of the CALLY index at risk of identifying oncological events at 2 years.

At the final follow-up, 112 patients were alive with the disease, and 50 patients died from their disease. The remaining 10 patients died of other causes. Overall, the 5-year DSS rate was 73.8% (95% confidence interval (CI), 66–80.1%). The CALLY index varied from 0.01 to 117.39. The average CALLY index was 15.54 (95% CI, 11.97–19.11). The CALLY index consists of serum albumin level, absolute lymphocyte count, and CRP. The average levels of albumin, CRP were 4.3 g/dL (95% CI, 4.19–4.34) and 1.32 (95% CI, 0.79–1.86). The average absolute lymphocyte count was 1847/μL (95% CI, 1759.4–1934.7). CRP was strongly correlated with the CALLY index (Spearman’s rho = −0.984, *p* < 0.0001). Serum albumin level had a moderate correlation (Spearman’s rho = 0.437, *p* < 0.0001). Absolute lymphocyte count had a weak correlation (Spearman’s rho = 0.268, *p* < 0.000372). Age, sex, and tumor depths were not associated with the CALLY index. Tumor grade was associated with the CALLY index. The median CALLY index in patients with low-grade STS was 13.76, while it was 3.99 in those with high-grade STS (*p* = 0.0064. chi-square test). Tumor size was weakly correlated with the CALLY index (Spearman’s rho = −0.293, *p* < 0.0001). Figure 1 shows the ROC curve analysis of the CALLY threshold for identifying the risk of identifying oncological events at two years. The threshold was 2.07, and the area under the curve (AUC) was 0.704 (95% CI, 0.621–0.787) (Figure 1).

The prognostic variables used to predict survival are shown in Table 2a,b. In the univariate analysis, age, tumor size, tumor site, tumor grade, perioperative Rx, and CALLY were prognostic variables. Multivariate analysis showed that tumor site, tumor size, and CALLY remained significant (Table 3).

**Figure 2:**
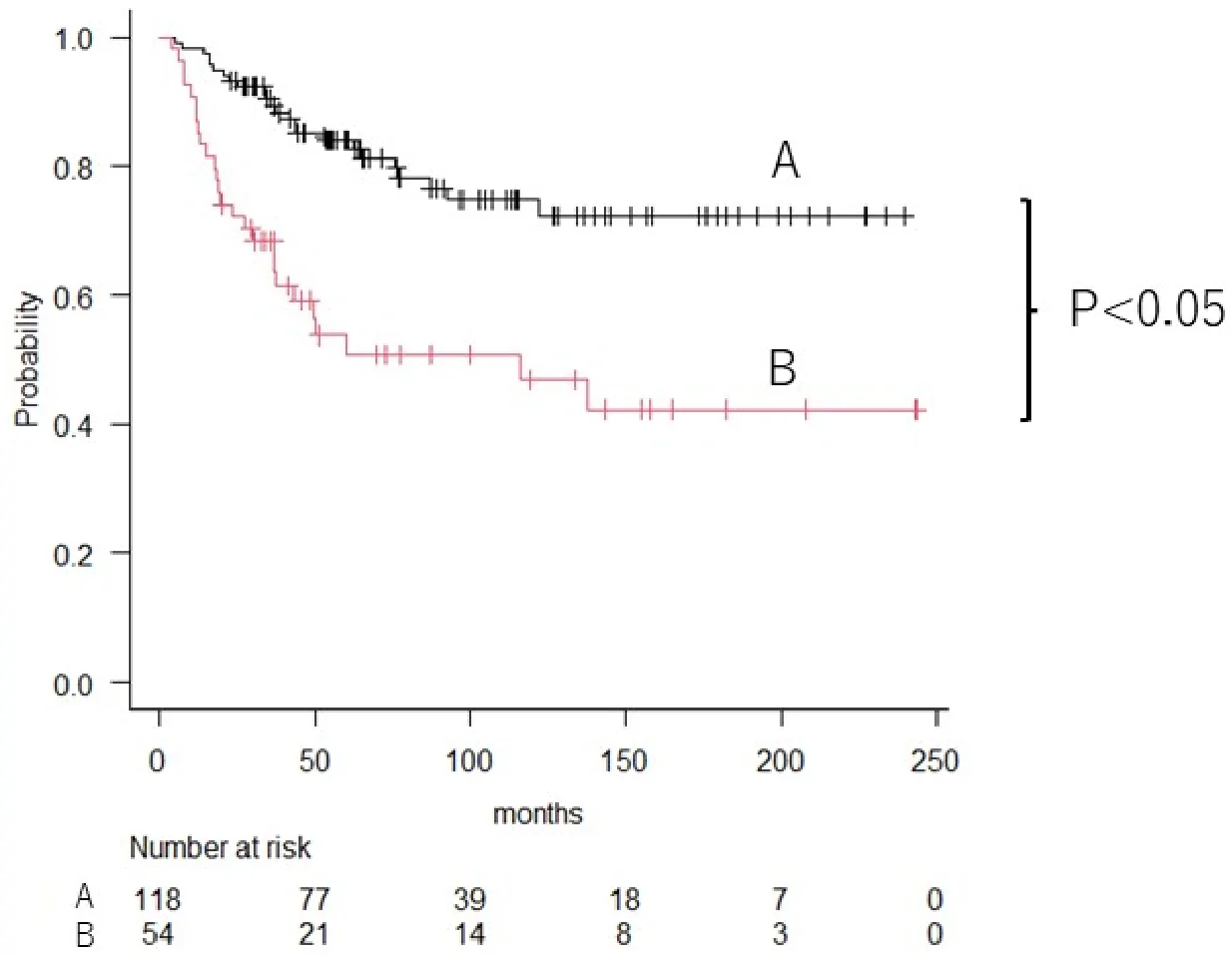
Kaplan–Meier curve showing disease-specific survival depending on the CALLY index. (A: CALLY index > 2.07, B: CALLY index < 2.07).

When we divided the patients into two groups according to the CALLY index of 2.07, the 5-year DSS in patients with a lower CALLY index (*n* = 54) was 50.9% (35.6–64.3), compared to 84% (75.5–89.8) in those with a higher CALLY index (*n* = 118) (*p* < 0.0001, log-rank test) (Figure 2).

Table 4 shows the relationship between the 5-years DSS and individual major histology according to the CALLY index. All 17 patients with MLPS had high CALLY indexes. Individually, CALLY index was a statistically significant prognostic variable for survival in UPS and DDLPS.

Local recurrence was observed as the initial event in 31 patients, and metastasis occurred in 52 patients. Local recurrence and metastasis were observed synchronously in 2 patients. The 5-year DFS rate was 55.5% (95% CI, 47.6–62.7) in all patients. Table 5 shows the prognostic variables for predicting DFS. In the univariate analysis, age, tumor size, tumor depth, tumor grade, and CALLY were prognostic variables. Multivariate analysis showed that age, tumor size, and CALLY remained significant (Table 6).

**Figure 3:**
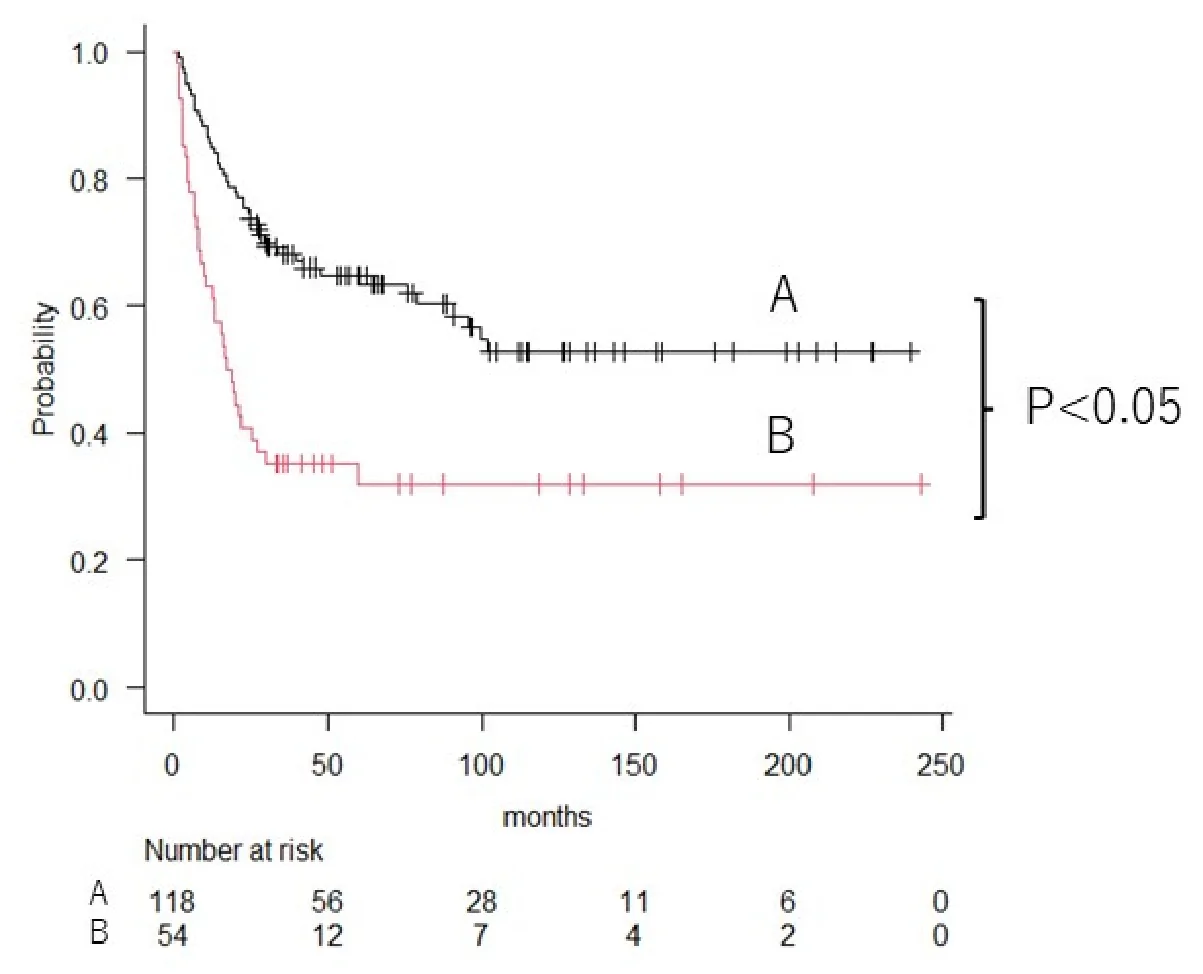
Kaplan–Meier curve showing the disease-free survival depending on CALLY index (A: CALLY index > 2.07, B: CALLY index < 2.07).

When we divided the patients into two groups according to the CALLY index of 2.07, the 5-year DFS in patients with a lower CALLY index (*n* = 54) was 35.2% (95% CI, 22.8–47.8), compared to 64.8% (95% CI, 55.2–72.9) in those with a higher CALLY index (*n* = 118) (*p* < 0.0001, log-rank test) (Figure 3).

#### 3.1.2. Relationship Between CALLY, PNI, and mGPS

PNI varied from 23.45 to 65.7, with an average of 51.91. ROC analysis at risk of identifying oncological events at 2 years showed that the CALLY index (AUC = 0.704) had a significantly higher AUC than the PNI (AUC = 0.577) (*p* = 0.0086).

The mGPS varied from 0 to 2. A total of 140 patients (81.4%) had a score of 0, 25 (14.5%) had a score of 1, and 7 (4.1%) had a score of 2. DSS and DFS improved in mGPS-dependent manner. The 5 years survival rate in patients with mGPS of 0, 1, and 2 was 82.2% (95% CI, 0.741–0.880), 40.4% (95% CI, 0.204–0.596), 28.6% (95% CI, 0.041–0.612), respectively. We compared ROC analysis between the CALLY index and CRP because mGPS consists of only three scores (0, 1, and 2). ROC analysis at risk of identifying oncological events at 2 years showed that there was no significant difference of AUC between CALLY index (AUC = 0.704) and CRP (AUC = 0.715).

**Figure 4:**
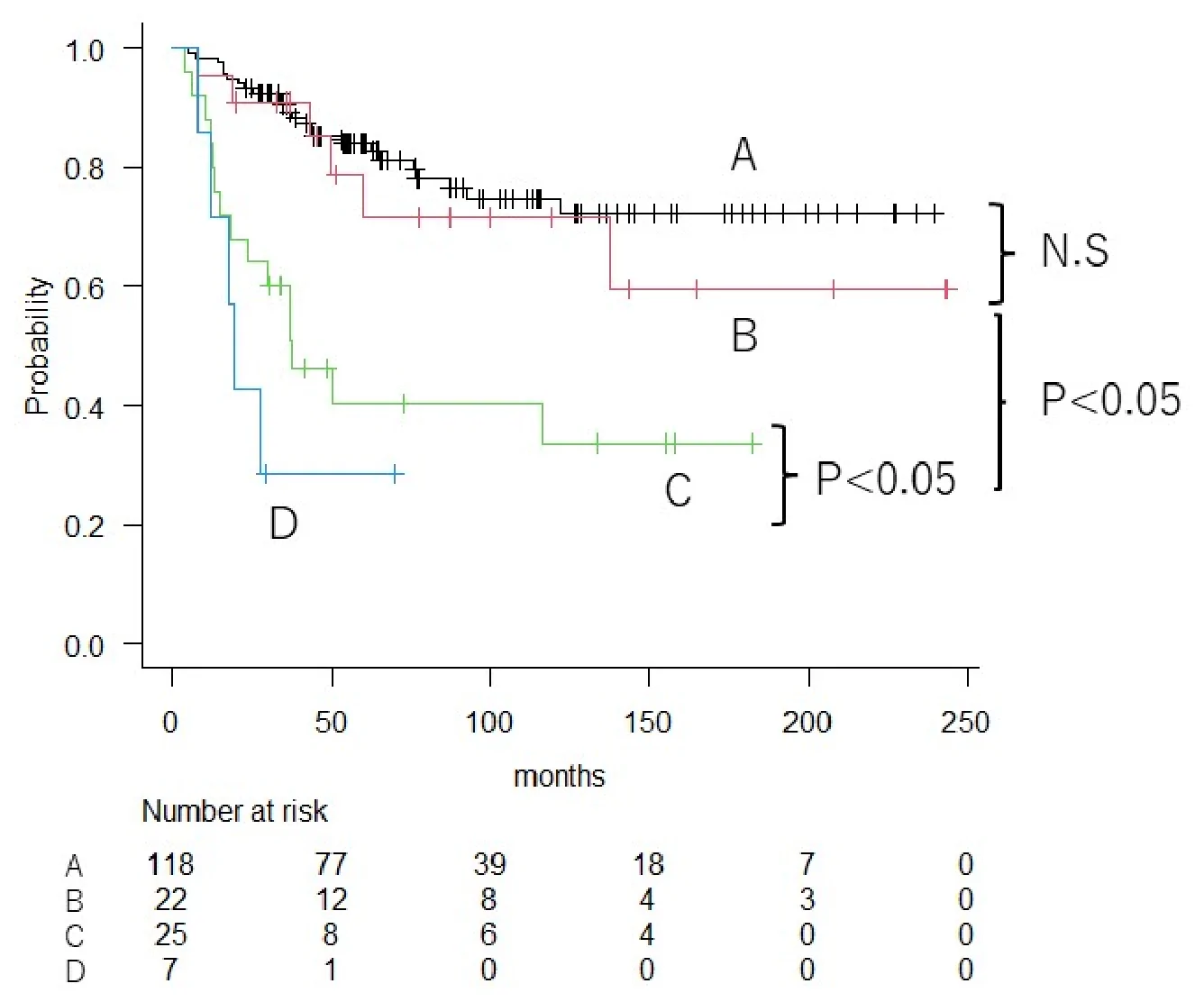
Kaplan–Meier curve showing disease-specific survival depending on the CALLY index and modified Glasgow prognostic score (mGPS). (A; CALLY index > 2.07 and mGPS of 0, B; CALLY index < 2.07 and mGPS of 0, C; CALLY index < 2.07 and mGPS of 1, D; CALLY index < 2.07 and mGPS of 2).

The mGPS of all 118 patients with a higher CALLY index was 0 (Table 7). The patients with mGPS of 1 and 2 had a significantly lower CALLY index (*p* < 0.0001, Chi-square test). When the Kaplan–Meier curve was created based on the CALLY index and mGPS, patients with a lower CALLY index and mGPS of 0 had better DSS and DFS than those with a lower CALLY index and mGPS of 1 or 2 (Figure 4). There were no significant differences in DSS and DFS between patients with a higher CALLY index and those with a lower CALLY index and an mGPS of 0. The 5-year survival rates in 118 patients with higher CALLY index and mGPS of 0 was 84% (95% CI, 75.5–89.8). The 5 year’s survival rate in patients with lower CALLY index and mGPS of 0, 1, and 2 was 71.5% (95% CI, 0.435–0.87.4), 40.4% (95% CI, 0.204–0.596), 28.6% (95% CI, 0.041–0.612), respectively.

The 5 years DFS rates in 118 patients with higher CALLY index and mGPS of 0 was 64.8% (95% CI, 0.552–0.729). The 5 years DFS rate in patients with lower CALLY index and mGPS of 0, 1, and 2 was 54.5% (95% CI, 32.1–72.4), 28% (95% CI, 0.124–0.460), 0%, respectively.

## 4. Discussion

The CALLY index, based on CRP, albumin, and lymphocyte levels, reflects the immune, nutritional, and inflammatory status of patients with cancer, which are crucial aspects in several types of cancer progression [16,17,18]. In the present study, tumor size and tumor grade were related to the CALLY index. Müller et al. developed CALLY in 2021 for patients with hepatocellular carcinoma (HCC) undergoing transarterial chemoembolization (TACE) [18]. They reported that the PNI was a favorable prognostic tool for survival stratification in patients with HCC that were undergoing TACE [19]. They hypothesized that CRP might play an additional component for prognostic score system in addition to PNI because it could reflect the inflammatory component of the tumor microenvironment and, thus, they developed the CALLY index, which combines the PNI with the CRP level [18]. Chen et al. pooled data from 18 studies which included a total of 7916 patients with digestive system cancers to comprehensively assess the impact of the CALLY index on the prognosis of patients with digestive system cancers [20]. They showed significant associations between the CALLY index and worse OS, DFS, relapse-free survival (RFS) and DFS in patients with digestive system cancers [20]. In this study, we demonstrated the usefulness of the CALLY index in predicting the survival of patients with primary STS. A lower CALLY index is significantly associated with poorer outcomes in several types of cancers. We showed that patients with a lower CALLY index had poor DDS and EFS. Additionally, we compared it with mGPS and PNI as the prognostic tools, including CRP, albumin, and lymphocyte counts. Interestingly, the mGPS was superior in predicting oncological outcomes because all patients with an mGPS of 1 or 2 had a lower CALLY index, and those with a higher CALLY index had an mGPS of 0. These results may be due to the significant role of CRP levels in the calculation of the CALLY index in primary STS. Actually, CRP was strong correlation with CALLY index using Spearman’s rank correlation analysis in this study. mGPS reflects the inflammatory condition, and CRP is a reliable marker for predicting oncological outcomes in STS [5,7,21,22]. Therefore, we suggested that mGPS may be more useful for predicting survival in patients with primary STS, compared to the CALLY index for predicting oncological events in patients with primary STS. The serum albumin level is a leading indicator of nutritional status and is likely to decrease secondary to a systemic inflammatory response [23,24]. In this study, serum albumin level was moderately correlated with the CALLY index. The utility of the CALLY index has been reported in digestive system cancers such as gastric, esophageal, and colorectal cancers [8,9,10,16,25,26,27]. Patients with these cancers are particularly affected by their nutritional status owing to their anatomical features. Malnutrition in patients with cancer is generally related to cancer-associated or cancer-induced weight reduction [28]. Cancer-associated weight reduction is caused by nutrient absorption disorders due to constriction or occlusion of the digestive tract, anorexia induced by anticancer drugs, or psychological stress [29]. The PNI is similar to the CALLY index [30,31]. Recent studies have demonstrated the high accuracy of the PNI in predicting treatment outcomes for various cancers, especially gastrointestinal cancer [30]. Immune system function plays a critical role in the efficacy of immune checkpoint inhibitors (ICIs), and serum albumin and lymphocyte levels are important indicators of immune system function. Therefore, the PNI has been reported to be a useful prognostic index for patients with gastrointestinal cancer who received ICI therapy [31]. Concerning STS, STS has often been regarded as an “immune cold” tumor category, clinical trials using immune checkpoint inhibitor monotherapy have not shown compelling clinical benefits, although combination therapy with dual checkpoint inhibitors or in combinations with other agents has yielded more promising results in de-differentiated LPS, UPS, angiosarcoma and alveolar soft-part sarcoma [32]. In the advanced stages of STS, nutrition and the immune system may be affected by chemotherapy, cachexia, and organ dysfunction [33,34]. Also, the utility of PNI was reported in patients with primary retroperitoneal STS, because these are prone to encase or abut vital viscera and major vessels, and patients with these STS are particularly affected by their nutritional status due to the similar cause of digestive system cancers [9,10]. Xe et al. reported 189 consecutive patients with retroperitoneal liposarcomas who underwent surgical treatment [9]. They showed that the low PNI group was observed with significantly worse median survival time and 5-year OS rates than that of the high PNI group (26 vs. 86 months, *p* < 0.001; 25.2% vs. 68.7%, *p* < 0.001; respectively). Garcia-Ortega et al. conducted a retrospective cohort study of adult 142 patients undergoing curative surgical resection of primary or recurrent retroperitoneal STS [10]. They showed that low PNI was associated with inferior 3-year OS (55% vs. 74%, *p*  =  0.008) and recurrent-free survival (44% vs. 63%, *p*  =  0.022). In this study, only three patients with retroperitoneal STS were included. Therefore, further studies on the utility of the CALLY index and PNI should be conducted in patients with advanced STS or primary retroperitoneal STS. Furthermore, the CALLY index was a statistically significant prognostic variable for survival in patients with UPS and DDLPS individually. Although there was no significant difference owing to the small number of the patients, the CALLY index may be useful in the patient with MFS. Therefore, further study targeting the patients with those STSs for which CALLY index can be used as a prognostic tool may be encouraged.

This study had some limitations. First, we focused on the preoperative assessment of the scoring system and its derivatives without evaluating postoperative changes. Second, we cannot focus deeply on individual histology owing to the limited number of patients in this study, although DSS seems to be worse for each histology with a lower CALLY index, except for LMS. Third, the optimal cut-off values for the nutritional indices and CALLY index may fluctuate with different sample sizes. In particular, absolute lymphocyte count may differ between study cohorts. Regarding the relationship between absolute lymphocyte count and STS, Brewster et al. reported that absolute lymphocyte count was significantly inversely associated with OS in 634 patients with musculoskeletal sarcomas [6]. The median absolute lymphocyte count was 1120/µL in their study. However, in another study, the median absolute lymphocyte count was 1770/µL and absolute lymphocyte count was not related with oncological outcome [35]. In addition, some patient-specific variables, such as alcoholism, medical comorbidities, smoking status, inflammation disease such as collagen disease, hepatitis, and access to healthy food, may affect the evaluation of the prognostic tools. Finally, the retrospective nature of this study is another limitation.

## 5. Conclusions

The CALLY index is a useful marker for predicting oncological outcomes in patients with primary STS. In particular, the CALLY index was a statistically significant prognostic variable for survival in patients with UPS and DDLPS individually. However, we suggest that the mGPS remains a reliable system for identifying patients with primary STSs who are at a high risk of death and relapse because we showed the significant difference for survival and event-free survival between the patients with mGPS of 1 and 2.

## Figures and Tables

**Table 1:** Patient’s characteristics.

Variable		*N*
Age (years)	Average	63
	Range	21–89
Sex	Male	100
	Female	72
Tumor size (cm)	Average	8.5
	Range	2–30
Depth	Superficial	39
	Deep	133
Grade	Low	23
	High	149
Tumor site	Thigh	66
	Leg	23
	Buttock	15
	chest wall	15
	upper arm	12
	Back	11
	Knee	6
	Others	24
Histology	UPS	39
	myxofibrosarcoma	36
	leiomyosarcoma	26
	MLPS	17
	DDLPS	15
	MPNST	9
	synovial sarcoma	8
	Others	22
Perioperative Cx	Yes	38
	No	134
Perioperative Rx	Yes	18
	No	154

Cx; chemotherapy, Rx: radiotherapy, UPS; Undifferentiated pleomorphic sarcoma, MLPS; myxoid liposarcoma, DDLPS; dedifferentiated liposarcoma, MPNST; malignant peripheral nerve sheath tumor.

**Table 2:** (**a**) Prognostic variables for disease-specific survival (qualitative variables); (**b**) Prognostic variables for disease-specific survival (quantitative data).

**(** **a** **)**				
**Variables**		***N***	**5Y-DSS (95% CI)**	***p*** **Value**
Sex	Male	100	75.8 (65.5–83.5)	0.45
	Female	72	70.7 (57.7–80.4)	
Depth	Superficial	39	69.9 (60.6–77.4)	0.243
	Depth	133	87.2 (71.9–94.5)	
Tumor site	Extremities	116	81.8 (72.9–88)	0.00101
	Trunk	58	56.4 (40.9–69.3)	
Grade	Low	23	94.1 (65–99.1)	0.0452
	High	149	70.8 (62.3–77.8)	
CALLY	>2.07	118	84 (75.5–89.8)	<0.0001
	<2.07	54	50.9 (35.6–64.3)	
Perioperative Rx	Yes	18	61.1 (67.1–81.8)	0.0298
	No	154	75.3 (67.1–85.8)	
Perioperative Cx	Yes	38	66.7 (48.8–79.4)	0.132
	No	134	76.1 (67.3–82.9)	
**(b)**				
**Variables**		**HR**	**95% CI**	***p*** ** Value**
Age	Years	1.023	1.001–1.045	0.0378
Size	Cm	1.119	1.069–1.171	<0.0001

Cx; chemotherapy, DSS; disease-specific survival, 95% CI; 95% confidential interval, HR: Hazard ratio.

**Table 3:** Multivariate analysis for predicting disease-specific survival.

Variables		HR	95% CI	*p*-Value
Age	Years	1.019	0.997–1.041	0.0971
CALLY index	>2.07	1		
	<2.07	2.361	1.28–4.356	0.006
Grade	High	1		
	Low	0.547	0.128–2.346	0.417
Tumor site	Extremities	1		
	Trunk	2.05	1.154–3.643	0.0144
Perioperative Rx	No	1		
	Yes	1.861	0.812–4.264	0.142
Tumor size	Cm	1.095	1.038–1.154	0.0008

HR: Hazard ratio, 95% CI; 95% confidential interval, Rx; Radiotherapy.

**Table 4:** The relationship between individual histology and survival.

Histology	CALLY Index	*N*	5-Years DSS	*p* Value
UPS	>2.07	18	86.6% (55.2–96.6)	0.0318
	<2.07	21	48.6% (25.2–68.5)	
Myxofibrosarcoma	>2.07	28	92% (71.5–98)	0.264
	<2.07	8	72.9% (27.6–92.5)	
Leiomyosarcoma	>2.07	16	64% (32.8–83.6)	0.95
	<2.07	10	70% (32.9–89.2)	
Myxoid liposarcoma	>2.07	15	85.6% (53.3–96.2)	0.642
	<2.07	2	not reached	
DDLPS	>2.07	9	88.9% (43.3–98.4)	0.0062
	<2.07	6	not reached	

UPS; undifferentiated pleomorphic sarcoma, DDLPS; dedifferentiated liposarcoma, DSS; disease-specific survival.

**Table 5:** (**a**) Prognostic variables for disease-free survival (qualitative variables); (**b**) Prognostic variables for disease-specific survival (quantitative data).

**(a)**				
**Variables**		***N***	**5Y-DFS (95% CI)**	***p*** **Value**
Sex	Male	100	53.9 (43.4–63.3)	0.882
	Female	72	57.5 (45.1–68.2)	
Depth	superficial	39	72.9 (55.3–84.5)	0.0247
	Depth	133	50.4 (41.5–58.7)	
Tumor site	Extremities	116	62.2 (52.5–70.4)	0.0019
	Trunk	56	41.5 (28.1–54.3)	
Grade	Low	23	89.3 (62.6–97.3)	0.0012
	High	149	50.3 (41.9–58.1)	
CALLY index	>2.07	118	64.8 (55.2–72.9)	<0.0001
	<2.07	54	35.2 (22.8–47.8)	
Perioperative Rx	Yes	18	38.1 (0.166–59.5)	0.0187
	No	154	57.8 (49.4–65.2)	
Perioperative Cx	Yes	134	44.6 (28.5–59.4)	0.196
	No	38	58.7 (49.6–66.7)	
**(b)**				
**Variables**		**HR**	**95% CI**	***p*** **Value**
Age	Years	1.024	1.008–1.041	0.00384
Size	cm	1.122	1.077–1.168	<0.0001

Cx; chemotherapy, DFS; disease-free survival, 95% CI; 95% confidential interval, HR: Hazard ratio.

**Table 6:** Multivariate analysis for predicting disease-free survival.

Variables		HR	95% CI	*p* Value
Age	Years	1.02	1.003–1.037	0.0182
CALLY index	>2.07	1		
	<2.07	1.903	1.197–3.028	0.0066
Grade	High	1		
	Low	0.346	0.106–1.128	0.0785
Tumor site	Extremities	1		
	Trunk	1.644	1.047–2.581	0.0308
Perioperative Rx	No	1		
	Yes	1.521	0.801–2.891	0.2
Depth	Deep	1		
	Superficial	0.858	0.462–1.593	0.627
Tumor size	Cm	1.099	1.049–1.152	<0.0001

HR: Hazard ratio, 95% CI; 95% confidential interval.

**Table 7:** The relationship between mGPS and CALLY index.

	mGPS of 0	mGPS of 1	mGPS of 2	*p* Value
CALLY > 2.07	118	0	0	<0.0001
CALLY < 2.07	22	25	7	

## Data Availability

The original contributions presented in this study are included in the article. Further inquiries can be directed to the corresponding author.

## References

[1] Clark M.A., Fisher C., Judson I., Thomas J.M. (2005). Soft-tissue sarcomas in adults. N. Engl. J. Med..

[2] Gronchi A., Miah A.B., Dei Tos A.P., Abecassis N., Bajpai J., Bauer S., Biagini R., Bielack S., Blay J.Y., Bolle S. (2021). Soft tissue and visceral sarcomas: ESMO-EURACAN-GENTURIS Clinical Practice Guidelines for diagnosis, treatment and follow-up. Ann. Oncol..

[3] Brennan M.F., Antonescu C.R., Moraco N., Singer S. (2014). Lessons learned from the study of 10,000 patients with soft tissue sarcoma. Ann. Surg..

[4] Bourcier K., Le Cense A., Tselikas L., Adam J., Mir O., Honore C., de Baere T. (2019). Basic knowledge in soft tissue sarcoma. Cardiovasc. Interv. Radiol..

[5] Nakamura T., Matsumine A., Asanuma K., Matsubara T., Sudo A. (2015). The value of the high-sensitivity modified Glasgow prognostic score in predicting the survival of patients with a soft-tissue sarcoma. Bone Jt. J..

[6] Brewster R., Purington N., Henry S., Wood D., Ganjoo K., Bui N. (2021). Evaluation of absolute lymphocyte count at diagnosis and mortality among patients with localized bone or soft tissue sarcoma. JAMA Netw. Open.

[7] Petrelli F., Barni S., Coinu A., Bertocchi P., Borgonovo K., Cabiddu M., Ghilardi M., Zaniboni A. (2015). The modified Glasgow prognostic score and survival in colorectal cancer: A pooled analysis of the literature. Rev. Recent Clin. Trials.

[8] Zhu D., Lin Y.D., Yao Y.Z., Qi X.J., Qian K., Lin L.Z. (2024). Negative association of C-reactive protein-albumin-lymphocyte index (CALLY index) with all-cause and cause-specific mortality in patients with cancer: Results from NHANES 1999–2018. BMC Cancer.

[9] Xue G.Q., Li C.P., Lv A., Wu J.H., Tian X.Y., Qiu H., Hao C. (2024). Prognostic Nutritional Index (PNI): A More Promising Nutritional Predictor for Patients Undergoing Surgery for Retroperitoneal Liposarcoma. Cancer Manag. Res..

[10] Garcia-Ortega D.Y., Lara-Robles N.O., Meléndez-Fernández A.P., Villavicencio-Valencia S.V., Irineo-Cerecedo D.R., Martinez-Valdovinos C., Bujanda-Sandoval D.A., Luna-Ortiz K. (2026). Preoperative prognostic nutritional index in prediction of surgical complications and oncological outcomes in retroperitoneal sarcoma. Updates Surg..

[11] Jiao Z., Liang C., Luo G., Liu M., Jiang K., Yang A., Liang Y. (2023). Prognostic Utility of Nutritional Risk Index in Patients with Head and Neck Soft Tissue Sarcoma. Nutrients.

[12] Spence S., Doonan J., Farhan-Alanie O.M., Chan C.D., Tong D., Cho H.S., Sahu M.A., Traub F., Gupta S., The MPGS Study Group (2022). Does the modified Glasgow prognostic score aid in the management of patients undergoing surgery for a soft-tissue sarcoma? An international multicentre study. Bone Jt. J..

[13] Nakamura T., Takenaka S., Outani H., Hagi T., Tamiya H., Imura Y., Asanuma K., Sudo A. (2024). The Combined Use of Inflammation Markers, Modified Glasgow Prognostic Score, and Sarculator Nomogram in Extremity Soft Tissue Sarcoma: A Multicenter Observational Study. Cancers.

[14] Hou T., Guo T., Nie R., Hong D., Zhou Z., Zhang X., Liang Y. (2020). The prognostic role of the preoperative systemic immune-inflammation index and high-sensitivity modified Glasgow prognostic score in patients after radical operation for soft tissue sarcoma. Eur. J. Surg. Oncol..

[15] Morhij R., Mahendra A., Jane M., McMillan D.C. (2017). The modified Glasgow prognostic score in patients undergoing surgery for bone and soft tissue sarcoma. J. Plast. Reconstr. Aesthet. Surg..

[16] Zhang Y., Liu W. (2026). The CALLY index in cancer research: A comprehensive review. Oncol. Rev..

[17] Li C., Zhou W., Zhao X., Sun Y., Zang J., Wang S. (2025). Meta-analysis of preoperative CALLY index for predicting the prognosis of cancer. Biomark. Med..

[18] Müller L., Hahn F., Mähringer-Kunz A., Stoehr F., Gairing S.J., Michel M., Foerster F., Weinmann A., Galle P.R., Mittler J. (2021). Immunonutritive scoring for patients with hepatocellular carcinoma undergoing transarterial chemoembolization: Evaluation of the CALLY index. Cancers.

[19] Müller L., Hahn F., Mähringer-Kunz A., Stoehr F., Gairing S.J., Foerster F., Weinmann A., Galle P.R., Mittler J., dos Santos D.P. (2021). Immunonutritive scoring in patients with hepatocellular carcinoma undergoing transarterial chemoembolization: Prognostic nutritional index or controlling nutritional status score?. Front. Oncol..

[20] Chen D., Ma Y., Li J., Wen L., Liu L., Su J., Wu J., Wang P., Zhang G., Huang C. (2025). Prognostic and clinicopathological significance of C-reactive protein-albumin-lymphocyte (CALLY) in patients with digestive system neoplasms: A systematic review and meta-analysis. World J. Surg. Oncol..

[21] Nakamura T., Matsumine A., Matsubara T., Asanuma K., Uchida A., Sudo A. (2012). Clinical significance of pretreatment serum C-reactive protein level in soft tissue sarcoma. Cancer.

[22] Wang X., Liu S., Zhao X., Fang E., Zhao X. (2019). The value of C-reactive protein as an independent prognostic indicator for disease-specific survival in patients with soft tissue sarcoma: A meta-analysis. PLoS ONE.

[23] Baracos V.E., Martin L., Korc M., Guttridge D.C., Fearon K.C.H. (2018). Cancer-associated cachexia. Nat. Rev. Dis. Prim..

[24] Eckart A., Struja T., Kutz A., Baumgartner A., Baumgartner T., Zurfluh S., Neeser O., Huber A., Stanga Z., Mueller B. (2020). Relationship of nutritional status, inflammation, and serum albumin levels during acute illness: A Prospective Study. Am. J. Med..

[25] Sakurai K., Kubo N., Hasegawa T., Nishimura J., Iseki Y., Nishii T., Inoue T., Yashiro M., Nishiguchi Y., Maeda K. (2024). Clinical significance of the CALLY index in patients with gastric cancer undergoing gastrectomy. World J. Surg..

[26] Bahardoust M., Shamohammadi M., Danesh N., Garavand A.A., Rezaei M.K., Alipour H., Haghmoradi M., Goodarzy B., Mousavie S.H., Tizmaghz A. (2025). Prognostic Value of CRP-Albumin-Lymphocyte (CALLY) Index in Colorectal Cancer Survival: A Multicenter Retrospective Cohort Study. J. Gastrointest. Cancer.

[27] Li J., Zhang S., Hu X., Huang T., Chen M. (2025). Correlation between the C-reactive protein (CRP)-albumin-lymphocyte (CALLY) index and the prognosis of gastric cancer patients after gastrectomy: A systematic review and meta-analysis. Surg. Today.

[28] Ottery F.D. (1994). Cancer cachexia: Prevention, early diagnosis, and management. Cancer Pract..

[29] Iida H., Tani M., Komeda K., Nomi T., Matsushima H., Tanaka S., Ueno M., Nakai T., Maehira H., Mori H. (2022). Superiority of CRP-albumin-lymphocyte index (CALLY index) as a non-invasive prognostic biomarker after hepatectomy for hepatocellular carcinoma. HPB.

[30] Yan L., Nakamura T., Casadei-Gardini A., Bruixola G., Huang Y.L., Hu Z.D. (2021). Long-term and short-term prognostic value of the prognostic nutritional index in cancer: A narrative review. Ann. Transl. Med..

[31] Zhang L., Ma W., Qiu Z., Kuang T., Wang K., Hu B., Wang W. (2023). Prognostic nutritional index as a prognostic biomarker for gastrointestinal cancer patients treated with immune checkpoint inhibitors. Front. Immunol..

[32] Zhu M.M.T., Shenasa E., Nielsen T.O. (2020). Sarcomas: Immune biomarker expression and checkpoint inhibitor trials. Cancer Treat. Rev..

[33] Evinç S.J., Akmercan Ç.E., Cebeci S., Birsin Z., Nazlı İ., Güren A.K., Işık S., Köylü B., Onur İ.D., Atasever T. (2026). Prognostic value of nutritional and inflammation-based scores in advanced soft tissue sarcoma treated with trabectedin: A multicenter study. Front. Oncol..

[34] Sabe H., Takenaka S., Kakunaga S., Tamiya H., Wakamatsu T., Nakai S., Takami H., Yamada Y., Okada S. (2025). Prognostic nutrition index as a predictive factor for overall survival in trabectedin-treated advanced soft tissue sarcoma. J. Orthop. Sci..

[35] Nakamura T., Hagi T., Asanuma K., Sudo A. (2022). Is lymphocyte C-reactive protein ratio useful for predicting survival in patients with non-metastatic soft tissue sarcoma?. Cancers.

